# Tendril Anatomy: A Tool for Correct Identification among Cucurbitaceous Taxa

**DOI:** 10.3390/plants11233273

**Published:** 2022-11-28

**Authors:** Naveed Abbas, Muhammad Zafar, Mushtaq Ahmad, Ashwaq T. Althobaiti, Mohamed Fawzy Ramadan, Trobjon Makhkamov, Yusufjon Gafforov, Khislat Khaydarov, Muhammad Kabir, Shazia Sultana, Salman Majeed, Tajalla Batool

**Affiliations:** 1 Department of Plant Systematics and Biodiversity Lab, Quaid-i-Azam University, Islamabad 45320, Pakistan; 2 Pakistan Academy of Sciences Islamabad, Islamabad 46000, Pakistan; 3 Department of Biology, College of Science, Taif University, P.O. Box 11099, Taif 21944, Saudi Arabia; 4 Department of Clinical Nutrition, Faculty of Applied Medical Sciences, Umm Al-Qura University, Makkah 21961, Saudi Arabia; 5 Department of Forestry and Landscape Design, Tashkent State Agrarian University, 2 A., Universitet Str., Kibray District, Tashkent 100700, Uzbekistan; 6 Mycology Laboratory, Institute of Botany, Academy of Sciences of Republic of Uzbekistan, 32 Durmon Yuli, Tashkent 100125, Uzbekistan; 7 AKFA University, 264 Milliy Bog Street, Tashkent 111221, Uzbekistan; 8 Faculty of Biology, Samarkand State University, Universitetsty Bulvvar Street-15, Samarkand 140104, Uzbekistan; 9 Department of Biological Sciences, Ex University of Sargodha Sub Campus Bhakkr, Thal University Bhakkar, Bhakkar 30000, Pakistan; 10 Department of Botany, University of Mianwali, Mianwali 42200, Pakistan

**Keywords:** anatomy, Cucurbitaceae, micromorphology, Parenchyma, vessel elements

## Abstract

This research examined the histological micro-structure of tendril vasculature in cucurbitaceous taxa. In this research, the tendril anatomy of 17 taxa of Cucurbitaceae categorized into seven genera, including *Cucumis* (five species), *Cucurbita* and *Luffa* (three species each), Citrullus and Momordica (two species each) while Lagenaria and Praecitrullus (one species each), collected from different areas of the Thal desert were examined via microscopic imaging to explore its taxonomic significance. Tendril transverse sections were cut with a Shandon Microtome to prepare slides. The distinctive characteristics of taxonomic value (qualitative and quantitative) include tendril and vascular bundle shape, variation in the number of vascular bundles, tendril diameter length, layers of sclerenchyma, and shape of collenchyma and epidermal cells. Tendril shapes observed are irregular, slightly oval-shaped, slightly C shaped, angular (4-angled, 6-angled, or polygonal), and star shaped. Quantitative measurements were taken to analyze the data statistically using SPSS software. *Cucurbita pepo* had a maximum tendril diameter length of 656.1 µm and a minimum in *Momordica balsamina of* 123.05 µm. The highest number of vascular bundles (12) were noticed in *Luffa acutangula* var*.amara. Angular* type was prominent in collenchyma, and irregular shape was dominant in sclerenchyma cells. A maximum of seven to nine sclerenchyma layers were present in *Lagenaria siceraria* and a minimum of two or three layers in *Cucumis melo* subsp. *agrestis*, *Cucumis melo* var. *flexuosus*, and *Cucumis melo* var.*cantalupensis.* Epidermis cells also show great variations with a rectangular shape being dominant. Statistical UPGMA dendrogram clustering of tendril vasculature traits shows that histological sections studied with microscopic techniques can be used to identify species and will play a vital role in future taxonomic and phylogenic linkages.

## 1. Introduction

The Cucurbitaceae family, or cucurbits, are most widely distributed in subtropical and tropical climates, with hotspots in West Africa, Southeast Asia, Mexico, and Madagascar [1]. Cucurbitaceous members (watermelons, cucumbers, luffas, pumpkin, courgettes, zucchini, and summer squash) are all edible and can be found growing across all continents. Around 800 species and 130 genera can be distributed worldwide [2]. In West Africa, this family is represented by 24 genera and 54 species [3]. In Pakistan, it has 33 species across 17 genera, including both domesticated (22 species) and wild species (11 species) [4]. The wild genera are *Coccinia, Lageneria, Luffa, Momordica,* and *Zehneria,* whereas cultivated include *Citrullus, Cucumis, Cucurbita, Cucumeropsis, Lagenaria, Telfairia*, and *Trichosanthes* [4]. However, Pakistan’s cucurbit species with high nutritious potential remain unexplored [5].

*Citrullus lanatus* is a succulent species belonging to the *Citrullus* genus and are desert vines and the only genus in the family with pinnatifid leaves. Watermelon (three varieties) and brown-seeded melon, both with bitter pulp, are members with solitary staminate flowers, tiny sepals, a basal corolla sectioned into five parts, and fleshy fruits. These are members of the subspecies *C. lanatus*, which is commonly cultivated in Pakistan. *Cucumis*, true melons, honey melons, and West Indian gherkins are all members of the twinning, tendril-bearing plants that belong to the *Cucumis* genus [6]. The leaves rarely split beyond the center. The fruits are smooth, green-lined, or hairy, with the appearance of a ground trailer. *Cucurbita* is a genus with approximately 20 species. The primary cultivated squash and pumpkin are four different *Cucurbita* species*: C. pepo, C. sativus, C. maxima,* and *C. pepo* var *cylindrica.* Ripe and immature fruit is the most important edible plant parts, although some species also consume seeds, flowers, roots, and even leaves. Cucurbitaceous species are not the only source of food, but are also used as a nutraceutical and pharmacotherapeutic potential [7]. The pepo’s sweet, delicately flavored, juicy flesh is eaten raw, frequently as a dessert. *Cucumis melo*, often known as sweet melon, is a fruit, not a vegetable.

Cucurbitaceous family members have lianous plant bodies, peculiar fleshy fruits (known as pepo), and a similar system of sex determination. The Cucurbitaceous species are primarily herbaceous plants with diverse pubescence and tuberous roots [2,8]. They are also physically distinguished by frequently angled stems and bicollateral vascular bundles that are frequently grouped in two concentric rings. The leaves are petiolate, exstipulate, alternating, often palmately veined, simple, or sedately complex, with extra-floral nectaries. The tendrils are lateral to the base of the petiole, usually one to four at each node, branching, simple-lobed, with a non-spiraling base [4].

Tendrils are an excellent example of convergent evolution because they have evolved numerous times among angiosperms. They can be found in lineages that are not closely related, such as Magnoliales and Asterales [9]. Tendrils are a perfect demonstration of their wide variation in morphology and ontogeny over the course of evolution. Tendrils can develop from modified twigs, pedicel, stipules, entire leaves, leaflets, leaf bracts, leaf apex, and inflorescences [10].

Environmental changes do not affect a plant’s anatomical characteristics [11]. Anatomical information was utilized to identify plant species, genera, and families. It is frequently employed in systematic identification, giving anomalous groupings a better classification position and illuminating relationship patterns that morphological traits may not have entirely conveyed. Nevertheless, it has been documented that cucurbit species in Pakistan can be distinguished by their plant morphology and anatomy [3]. Several [12,13,14] researchers describe anatomical characters of Cucurbitaceous species however, tendril micromorphology is not visualized [15]. Plant cell and tissue distributions such as sclerenchyma, vascular bundles, and other anatomic traits have been described at various systematic levels for species delimitation [16]. The presence of trichomes on the lower surface of the leaves, which reflects significant taxonomic relevance, is well recognized in the Cucurbitaceae [17]. Comparative and systematic studies on the anatomy of the various vegetative organs (root, stem, and leaf) of the species of the Cucurbitaceae family were carried out [13,14,18,19].

Thus, to better understand the systematic relationships, there is a need to study different field characteristics, like the anatomical features of tendrils involved in plant taxonomy. This study aimed to analyze and describe the significant tendril micromorphological traits which will contribute to the anatomy of the Cucurbitaceae and provide features for accurate species identification and their taxonomic implications.

## 2. Results

**Figure 1:**
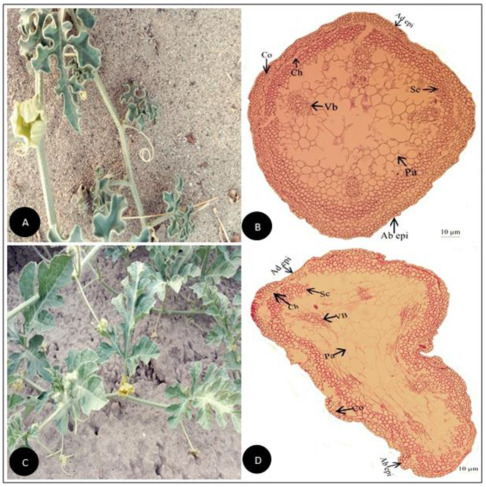
(**A**) Field pictorial view of *Citrullus colocynthis* (L.) Schrad. (**B**) Tendril cross-section of *Citrullus colocynthis* (L.) Schrad. (Scale bar = 10 µm) (**C**) Field pictorial view of *Citrullus lanatus* (Thunb.) Matsum. & Nakai (**D**) Tendril cross section of *Citrullus lanatus* (Thunb.) Matsum.& Nakai (Scale bar = 10 µm).Ad epi, Adaxial epidermis; Ab epi, Abaxial epidermis; Co, Collenchyma; Ch, Chlorenchyma; Sc, Sclerenchyma; Pa, Parenchyma; Vb, vascular bundle.

**Figure 2:**
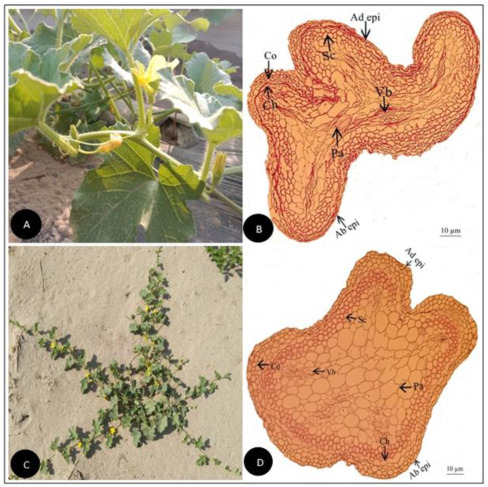
(**A**) Field pictorial view of *Cucumis melo* L. (**B**) Tendril cross-section of *Cucumis melo* L. (Scale bar = 10 µm) (**C**) Field pictorial view of *Cucumis melo subsp. agrestis* (Naudin) Pangalo (**D**) Tendril cross-section of *Cucumis melo subsp. agrestis* (Naudin) Pangalo (Scale bar = 10 µm). Ad epi, Adaxial epidermis; Ab epi, Abaxial epidermis; Co, Collenchyma; Ch, Chlorenchyma; Sc, Sclerenchyma; Pa, Parenchyma; Vb, vascular bundle.

**Figure 3:**
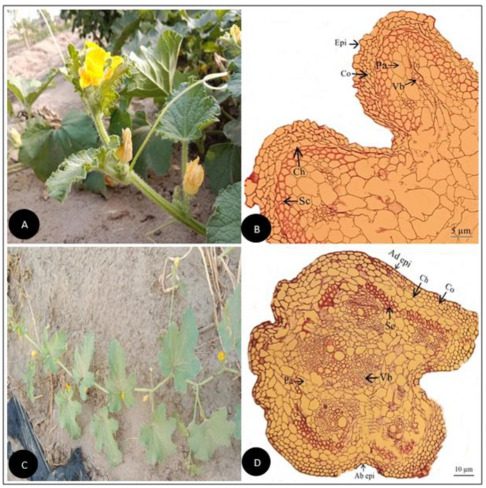
(**A**) Field pictorial view of *Cucumis melo var. flexuosus* (L.) Naudin (**B**) Tendril cross-section of *Cucumis melo* var. *flexuosus* (L.) Naudin (Scale bar = 10 µm) (**C**) Field pictorial view of *Cucumis melo var.cantalupensis* Naudin (**D**) Tendril cross-section of Cucumis melo var.cantalupensis Naudin (Scale bar = 10 µm). Ad epi, Adaxial epidermis; Ab epi, Abaxial epidermis; Co, Collenchyma; Ch, Chlorenchyma; Sc, Sclerenchyma; Pa, Parenchyma; Vb, vascular bundle.

**Figure 4:**
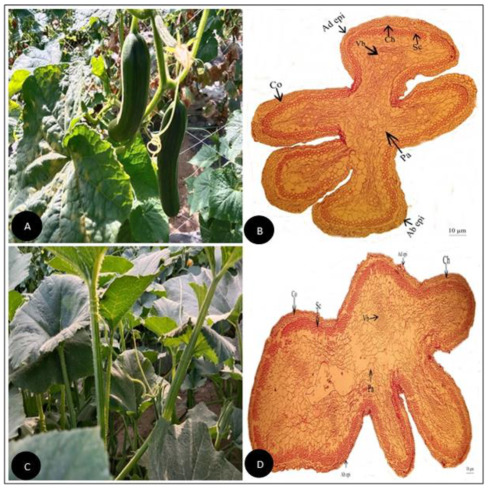
(**A**) Field pictorial view of *Cucumis sativus* L. (**B**) Tendril cross-section of *Cucumis sativus* L. (Scale bar = 10 µm) (**C**) Field pictorial view of *Cucurbita maxima* Duchesne (**D**) Tendril cross-section of *Cucurbita maxima* Duchesne (Scale bar = 10 µm). Ad epi, Adaxial epidermis; Ab epi, Abaxial epidermis; Co, Collenchyma; Ch, Chlorenchyma; Sc, Sclerenchyma; Pa, Parenchyma; Vb, vascular bundle.

**Figure 5:**
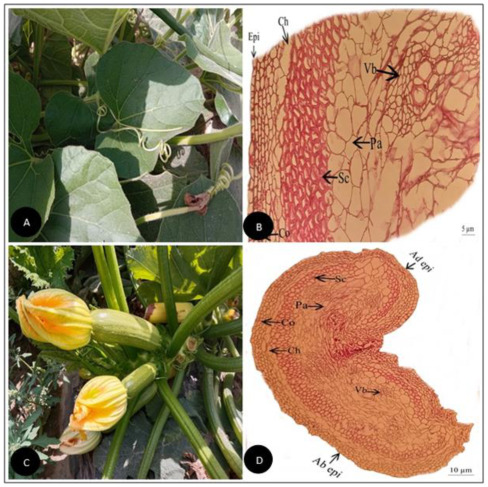
(**A**) Field pictorial view of *Cucurbita pepo* L. (**B**) Tendril cross-section of *Cucurbita pepo* L. (Scale bar = 5 µm) (**C**) Field pictorial view of *Cucurbita pepo var. cylindrica* (**D**) Tendril cross-section of *Cucurbita pepo var. cylindrica* (Scale bar = 10 µm). Ad epi, Adaxial epidermis; Ab epi, Abaxial epidermis; Co, Collenchyma; Ch, Chlorenchyma; Sc, Sclerenchyma; Pa, Parenchyma; Vb, vascular bundle.

**Figure 6:**
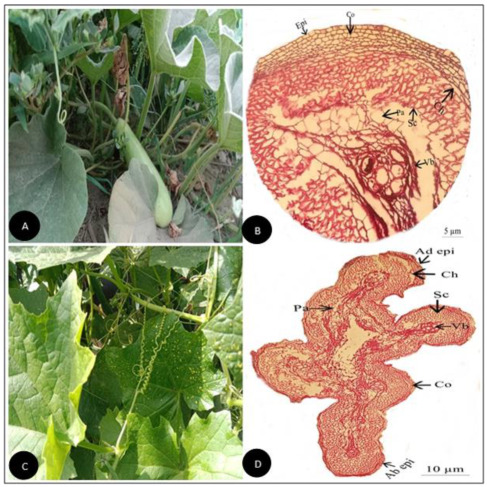
(**A**) Field pictorial view of *Lagenaria siceraria* (Molina) Standl. (**B**) Tendril cross-section of *Lagenaria siceraria* (Molina) Standl. (Scale bar = 5 µm) (**C**) Field pictorial view of *Luffa acutangula* (L.) Roxb (**D**) Tendril cross-section of *Luffa acutangula* (L.) Roxb (Scale bar = 10 µm). Ad epi, Adaxial epidermis; Ab epi, Abaxial epidermis; Co, Collenchyma; Ch, Chlorenchyma; Sc, Sclerenchyma; Pa, Parenchyma; Vb, vascular bundle.

**Figure 7:**
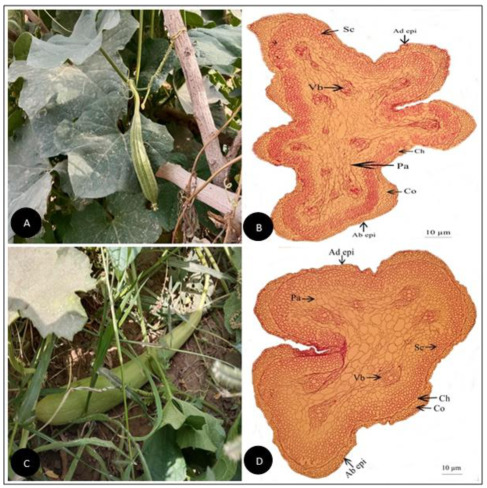
(**A**) Field pictorial view of *Luffa acutangula* var. *amara* C.B.Clarke (**B**) Tendril cross-section of *Luffa acutangula var. amara* C.B.Clarke (Scale bar = 10 µm) (**C**) Field pictorial view of *Luffa cylindrica*(L.) M.Roem (**D**) Tendril cross-section of *Luffa cylindrica*(L.) M.Roem (Scale bar = 10 µm). Ad epi, Adaxial epidermis; Ab epi, Abaxial epidermis; Co, Collenchyma; Ch, Chlorenchyma; Sc, Sclerenchyma; Pa, Parenchyma; Vb, vascular bundle.

**Figure 8:**
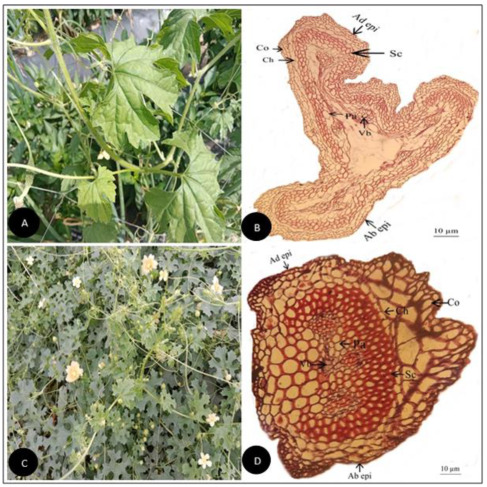
(**A**) Field pictorial view of *Momordica charantia* L. (**B**) Tendril cross-section of *Momordica charantia* L. (Scale bar = 10 µm) (**C**) Field pictorial view of *Momordica dioica* Roxb. ex Willd. (**D**) Tendril cross section of *Momordica balsamina L.* (Scale bar = 10 µm). Ad epi, Adaxial epidermis; Ab epi, Abaxial epidermis; Co, Collenchyma; Ch, Chlorenchyma; Sc, Sclerenchyma; Pa, Parenchyma; Vb, vascular bundle.

**Figure 9:**
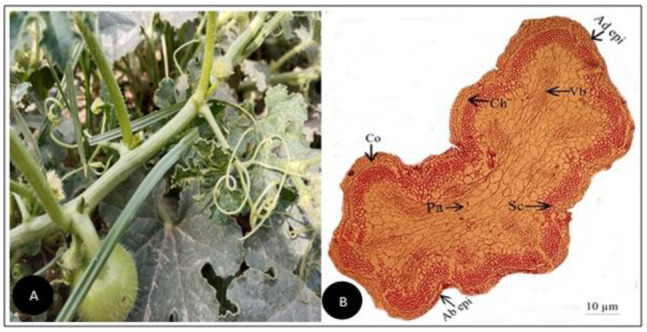
(**A**) Field pictorial view of *Praecitrullus fistulosus* (Stocks) Pangalo (**B**) Tendril cross section of *Praecitrullus fistulosus* (Stocks) Pangalo (Scale bar = 10 µm). Ad epi, Adaxial epidermis; Ab epi, Abaxial epidermis; Co, Collenchyma; Ch, Chlorenchyma; Sc, Sclerenchyma; Pa, Parenchyma; Vb, vascular bundle.

Tendril anatomical traits observed in the current investigation were outline, vascularization forms, vessel elements, collenchyma, chlorenchyma, sclerenchyma, as well as parenchyma tissues as illustrated (Figure 1, Figure 2, Figure 3, Figure 4, Figure 5, Figure 6, Figure 7, Figure 8 and Figure 9). The summarized qualitative and quantitative results are given in (Table 1, Table 2, Table 3 and Table 4). Observing their recorded taxonomic evaluation, cucurbitaceous taxa showed significant size and shape variation (Figure 1, Figure 2, Figure 3, Figure 4, Figure 5, Figure 6, Figure 7, Figure 8 and Figure 9).

**Figure 10:**
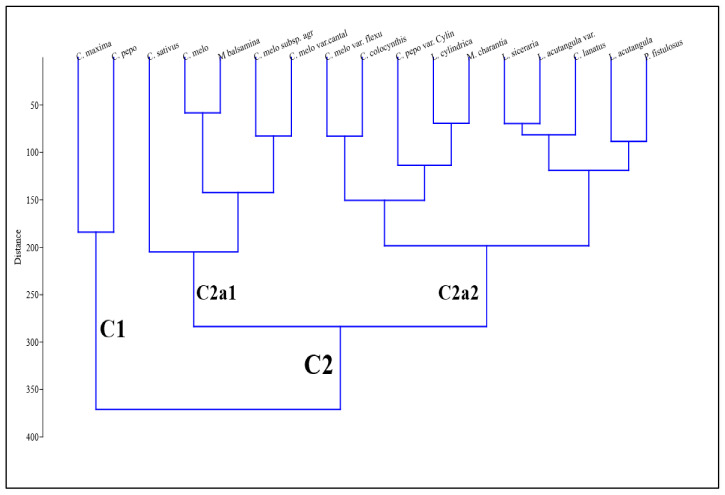
Cluster groupings via dendrogram of Cucurbitaceous taxa based on tendril features.

The UPGMA clustering dendrogram for Cucurbitaceous taxa is presented in Figure 10.

Seventeen taxa of Cucurbitaceae fall into two major clusters based on the difference in qualitative features. Similarity relationships among different Cucurbitaceous species were explored using UPGMA clustering using tendril anatomical characters. The UPGMA phenogram shows two main clusters, C1 and C2. The first principle cluster, C1, represents sections *C. maxima* and *C. pepo*. The second cluster C2 further divided into two sub-clusters comprising C2a1 of 5 species in which *C. melo* and *C. balsamina* were closely related based on Euclidean distance mapping. The second sub-cluster, C2a2, represents ten species, among which, based on Euclidean distance *L. cylindrica* and *M. charantia* was placed at the minimum distance in this sub-cluster, showing the similarity in tendril qualitative features.

### Identification Keys Based on Cucurbitaceous Tendril Features

1 + Lamellar Collenchyma …………………………………………………...........................**2  **− Angular Collenchyma…….……………………………………………………….............**5**2 + Vascular bundle with round shape, irregular tendril outline…...…….............**L. siceraria**- Lamellar and angular collenchyma………………….…………………………..…...……**3**3 + Irregular vascular bundle, 6-angled tendril outline………………………………..**C. pepo**- Oval and irregular vascular bundle………………..………………………………..……..**4**4 + C shaped tendril shape…………………………………..……….**C. pepo var. cylindrica** - Angular collenchyma cell layers……..…………………………………...……………….**5**5 + Tendril outline oval, vascular bundle slightly oval……..……………..……**C. colocynthis** - Subsidiary type vascular bundles………………………………………………………….**6**6 + V shaped tendril, elliptical shape vascular bundle…………….………………...**C. lanatus**- Irregular tendril outline…………...……………………………………………………….**7**7 + Subsidiary type vascular bundle, rectangular epidermal cells……………………...**C. melo**- Polygonal sclerenchyma cells……………………………….……………………………..**8**8 + 4-angled tendril outline, irregular vascular bundle………………..**C. melo subsp. agrestis**- Irregular tendril shape…………..………………………………………………................**9**9 + Tetragonal sclerenchyma cells…………………….………………**C. melo var. flexuosus  -**Triangular to polygonal sclerenchyma cells………..………………………………………**10**10 + Central vascular bundle type..………......................................**C. melo var. cantalupensis**- Elliptical and irregular vascular bundle shape…………………………………………...**11**11 + Star shape tendril outline……………………………………………………….**C. sativus**- Rectangular epidermal cells, irregular tendril…………………….……………………**12**12 + Dumbbell type vascular bundle………………………………………………..**C. maxima**- Rounded vascular bundle shape………………………..………………………………**13**13 + Irregular with hollow pith tendril sape……………………..…………..…..**L. acutangula**- Rounded to oval vascular bundle shape………….……………………………………**14**14 + Polygonal tendril outline…………………………………...… **L. acutangula var.amara**  - Hexagonal tendril outline.…………………………………………**15**15 + Hexagonal to polygonal chlorenchyma cells………………………….……**L. cylindrica**- 4-angled tendril, rectangular to polygonal chlorenchyma cells………………………**16**16 + Central type vascular bundle, tetragonal sclerenchyma cells………………**M. balsamina**- Pentagonal chlorenchyma cells…………………………………………………………**17**17 + Rounded and elliptical vascular bundle type……………………………**M. charantia**- Rectangular epidermal cells, subsidiary vascular bundle type…………………………1818 + Polygonal slightly U shaped tendril outline………………………………….**P. fistulosus**

## 3. Discussion

**Figure 11:**
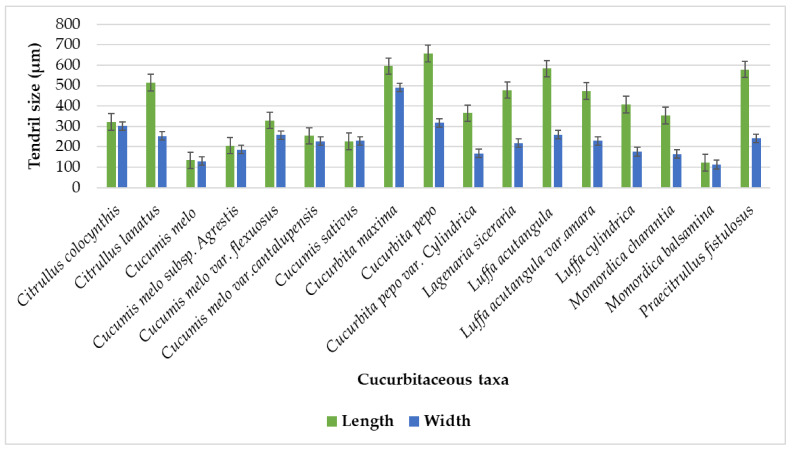
Mean tendril size variations among Cucurbitaceous taxa.

Various studies described morpho-anatomical features to classify Cucurbitaceous species [13,14]. Different researchers have carried out the tendril anatomical investigation of a few species of Cucurbitaceae [20] and studied the anatomical characteristics of some species of the genus cucurbita, like flower stalk, petiole, and stem, and examined some tendril characteristics. Leaf anatomy of Cucurbitaceous species has been briefly mentioned by [21], but no information is available on the tendril anatomy of Cucurbitaceous taxa, so this study elaborates tendril histology of 12 Cucurbitaceous taxa to find out taxonomic markers for their correct identification. The tendril’s shape was four and five angled furrows in *Momordica charantia* and *Cucumis sativa* [22], which shows similarities with current results. While current studies show tendrils outlined in transverse view in different cucurbitaceous taxa are mostly irregular, slightly oval-shaped, slightly C shaped, angular (four-angled, six-angled, or polygonal), and star-shaped (Table 1). The maximum tendril length was observed in *Cucurbita pepo* (656.1 µm) and minimum was observed in *Momordica balsamina* (123.05 µm) as shown in Figure 11. Whereas the maximum width of tendril was noted in *Cucurbita maxima* (489.6 µm) and minimum in *Momordica balsamina* (112.95 µm).

**Figure 12:**
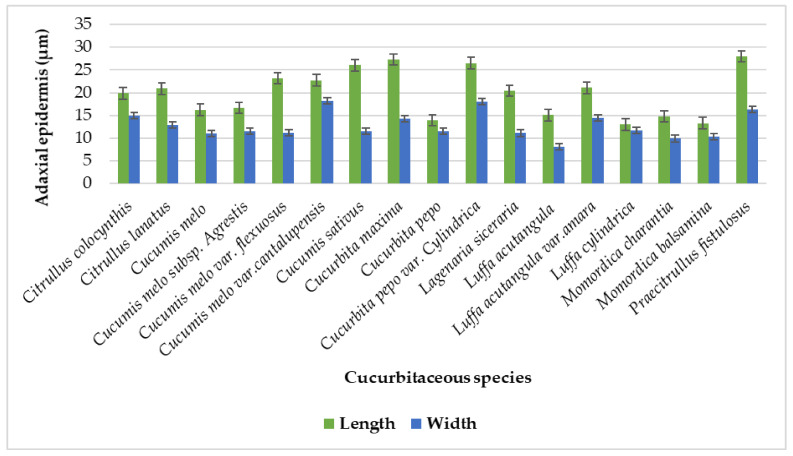
Graphical representation for epidermal cell size on the Adaxial surface of tendril.

There was a single-layered epidermis present in the tendril histological section and it was predominantly irregular in shape while oval-shaped cells were recorded [22]. However, [23] elaborates on rectangular cells whereas our findings show square, oval, isodiametric, irregular, pentagonal, hexagonal, and polygonal types. Significant variation in the tendril micromorphology of Cucurbitaceous taxa was observed on both the adaxial and abaxial epidermal sides. There was variation in the epidermal cell length and width of the studied species (Table 3). The largest cell lengthwise was noted on the adaxial side in *Praecitrullus fistulosus* (28 µm) and the smallest in *Luffa cylindrica* (13 µm). The maximum epidermal cell width was noted in *Cucumis melo* var.*cantalupensis* (18.2 µm) and minimum in *Luffa acutangula* (8.05 µm) as shown in Figure 12.

**Figure 13:**
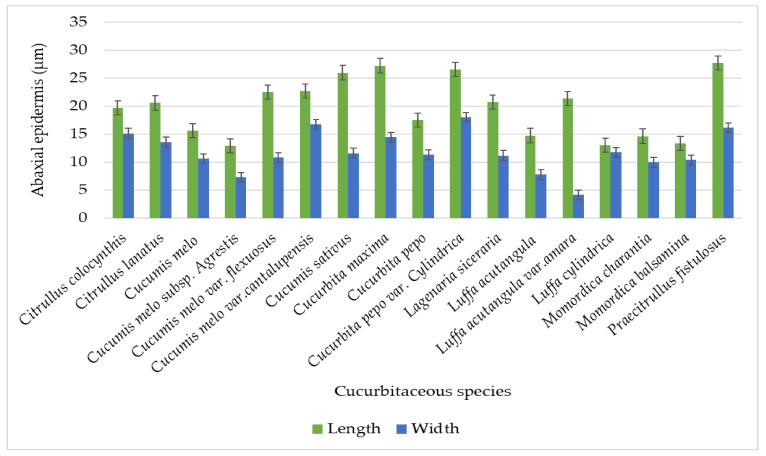
Graphical representation for epidermal cell size on the abaxial surface of the tendril.

Correspondingly, the largest cell length was calculated along the abaxial in *Praecitrullus fistulosus* (27.65 µm) and the shortest in *Cucumis melo* subsp. *agrestis* (12.85 µm). The cell width was observed to be maximum on the abaxial surface in *Cucurbita pepo* var. *cylindrica* (18 µm) and minimum in *Luffa acutangula* var. *amara* (4.1 µm). Layers of sclerenchyma and chlorenchyma (both one to eight layers) and collenchyma cells (two to six layers). However, [22] mentioned some variations in sclerenchyma two to nine, chlorenchyma one to three and collenchyma two to six as mentioned in Figure 13.

**Figure 14:**
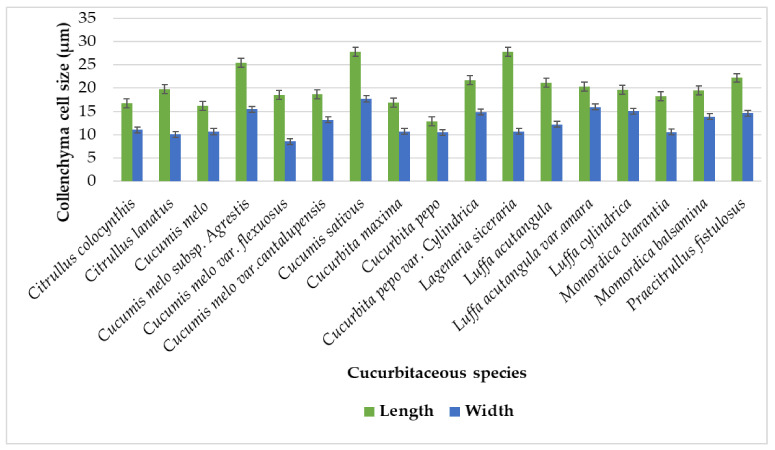
Graphical Representation of collenchyma length and width on the surface of tendril.

Angular collenchyma cells were observed from Iraq by [23] among Cucurbitaceae species from Iraq, while angular lamellar types were examined in this study (Table 1). In previous studies, two to four layers of collenchyma were present [22], while present measurements revealed a distinct continuous layering of cells below the epidermis with two to six layers of collenchyma (Table 2). The maximum layers were present in *Cucurbita maxima* and *Luffa acutangula* var.*amara* both having six layers, while the minimum number of layers was present in *Citrullus lanatus*, with two or three layers. Collenchymatous cell size showing maximum length in in *Cucumis sativus* (27.85 µm) while minimum length was in *Cucurbita pepo* (12.85 µm). Whereas the largest width was calculated for *Cucumis sativus* (17.7 µm) the lowest width was for *Cucumis melo* var. *flexuosus* (8.55 µm) as illustrated in Figure 14.

**Figure 15:**
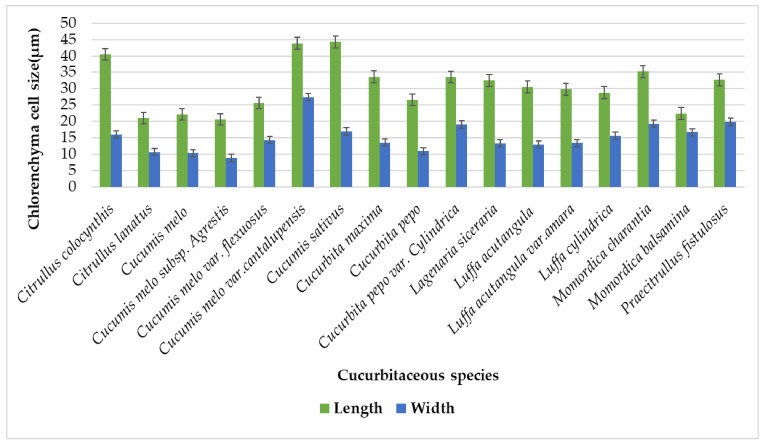
Graphical representation of chlorenchyma length and width on the surface of tendril.

There were mostly two or three layers of chlorenchyma in tendrils of Cucurbitaceae species [22], while in recent studies, a single layer of chlorenchyma cells lies beneath collenchyma but in some species, more than single layers noticed two or two to three layers (Table 2). Maximum chlorenchymateous layers were present in *Lagenaria siceraria* in two to three layers. Different shapes of chlorenchyma cells were inspected, such as rectangular, irregular, pentagonal, hexagonal, and polygonal (Table 1). The chlorenchymateous cell size range from largest lengthwise was measured in *Cucumis sativus* (44.25 µm) while the shortest was in *Cucumis melo* subsp. *agrestis* (20.6 µm). Likewise, the largest cell widthwise was seen in *Cucumis melo* var. *cantalupensis* (27.45 µm), and the smallest cell widthwise was observed in *Cucumis melo* subsp. *agrestis* (8.9 µm) Figure 15.

**Figure 16:**
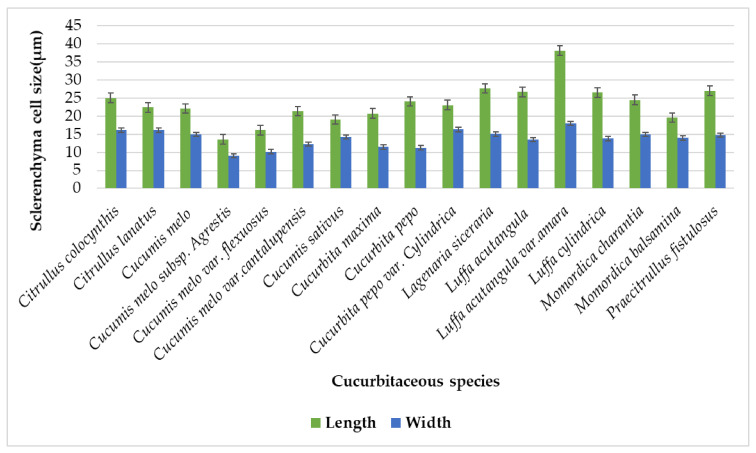
Graphical representation of sclerenchyma length and width on the surface of the tendril.

Species like *Citrullus colocynthis* and *Citrullus lanatus* have continuous sclerenchyma cells, similar to our findings. All the studied species illustrated the continuous sclerenchymatous cells except *Cucumis melo* var *catanlupensus, Cucurbita pepo* var *cylindrica,* and *Momordica charantia*, discontinuous sclerenchymatous cells were recorded in these species. The continuous layer of sclerenchymatous cells that makes up the tendril acts as an anchor and requires it to be robust enough to hold the weight of the plant and its fruits, especially when it is rising [22]. The most prominent and darkly stained layers of sclernchyma cells were just below the chlorenchyma cell layers. Sclerenchyma cell layers range from a minimum of two to three layers in three species of *Cucumis melo* subsp. *agrestis*, *Cucumis melo* var. *flexuosus*, and *Cucumis melo* var. *cantalupensis,* while the maximum number of layers was noticed in *Lagenaria siceraria* seven to nine layers, Table 2. Shapes of sclerenchyma cells are dissimilar in tendrils of studied plant species. Mainly, sclerenchyma was perceived as irregular, trigonal, tetragonal, hexagonal, and polygonal shaped, Table 1. There were variations in sclerenchyma cell size ranges, maximum cell sizes lengthwise were seen in *Luffa acutangula* var. *amara* (38.05 µm) compared to minimum cell size lengthwise in *Cucumis melo* subsp*. agrestis* (13.55 µm). The maximum width of sclerenchyma cells was noted in *Luffa acutangula* var. *amara* (17.95 µm), while minimum was observed in *Cucumis melo* subsp. *agrestis* (9 µm) Figure 16.

**Figure 17:**
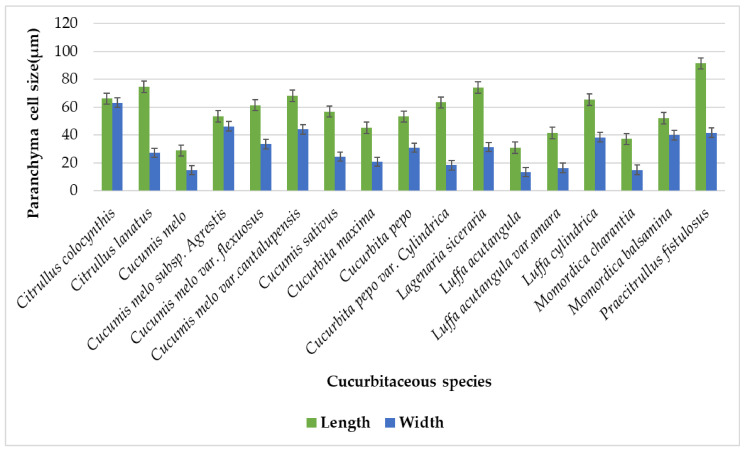
Graphical representation of parenchyma length and width on the surface of the tendril.

In earlier studies, different parenchyma cells were irregular, pentagonal, and polygonal (Table 1), butt angular parenchyma cells were also recorded [23]. In the current studies, parenchymatous cells were present in all species, mostly occupying the pith region in tendrils. The number of parenchyma cell layers differed in all studied species in Table 2. The maximum parenchyma layers were present in *Cucumis sativus* six-layers, whereas the minimum was observed in Luffa acutangula var. amara of two-layers. Parenchyma cells were the largest cells in size present in tendrils. The largest cell lengthwise is Praecitrullus fistulosus (91.55 µm), while mini cells were present in *Cucumis melo* (29 µm). The sizeable cells, widthwise, are present in *Citrullus colocynthis* (63.35 µm), and compact cells widthwise were existingin *Luffa acutangula* (13.5 µm) Figure 17.

**Figure 18:**
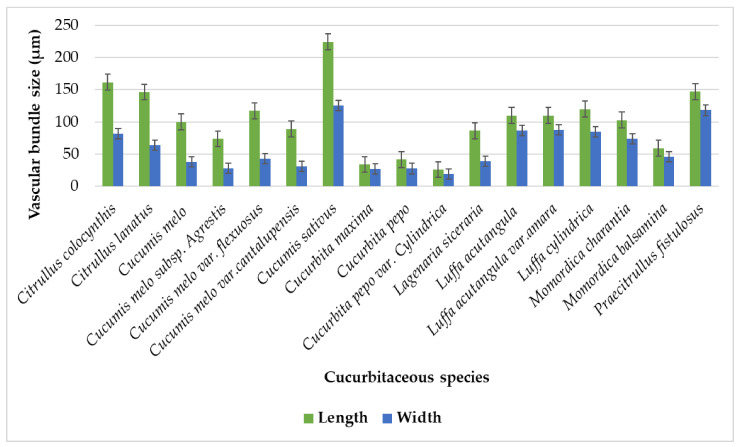
Graphical illustration showing vascular bundle size of tendril.

Vascular bundles were mainly arranged in a subsidiary manner, and few were centrally arranged in the case of *Cucumis melo* var. *cantalupensis* and *Momordica charantia,* Table 1. Bicollateral-types of vascular bundles were present in tendrils. Various writers have reported this feature in the Cucurbitaceae, which is constant across the analyzed taxa [3,4,14,24]. Their shapes vary from oval, elliptical, rounded, irregular, and dumbbell, Table 1. Each studied species has a different number of vascular bundles (Table 2). The maximum number of vascular bundles was detected in *Luffa acutangula var. amara,* having 12 vascular bundles, while the minimum number of vascular bundles was in *Momordica balsamina,* having three vascular bundles. Vascular bundles also vary in size. The largest vascular bundle lengthwise was present in *Cucumis sativus* (224.25 µm), while the smallest was observed in *Cucurbita pepo* var. *cylindrica* (26.1 µm). The largest vascular bundle widthwise was observed in *Cucumis sativus* (125.3 µm), and the smallest vascular bundle was present in *Cucurbita pepo* var. *cylindrica* (19.35 µm) Figure 18.

**Figure 19:**
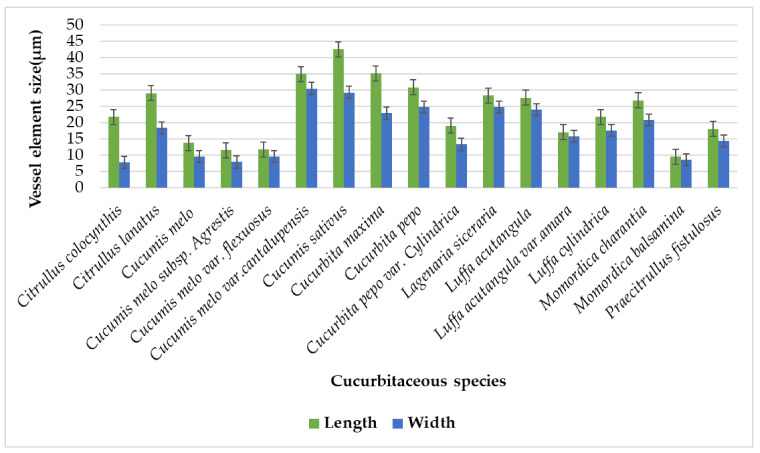
Graphical variations among tendril vessel elements.

There was no record found about the vessel elements of the vascular bundle previously, while the current study showed a distinct number of vessel elements in the xylem of the studied species’ tendrils. Their numbers and size vary from species to species. The highest number of vessel elements were present in *Cucumis sativus,* of about 15, and the lowest number of vessel elements existed in *Cucumis melo* var. cantalupensis, at around four elements (Table 2). The biggest vessel element lengthwise was found in *Cucumis sativus* (42.55 µm), while the smallest vessel element lengthwise was present in *Momordica balsamina* (9.5 µm). The largest vessel element widthwise was observed in *Cucumis melo* var. *cantalupensis* (30.45 µm); meanwhile, the shortest was analyzed in *Citrullus colocynthis* (7.82 µm) Figure 19.

No tendril anatomical data were found about *Luffa aegyptiaca*. However, the species was used against hydrocarbon-contaminated soil through rhizoremediation, and chemical analysis of this species was also carried out in research papers [25]. In Western Africa, three genera of the Cucurbitaceae family, e.g., *Momordica*, *Luffa,* and *Trichosanthes,* were studied for their foliar epidermis and tendril morphology. The significant differences in their leaf and tendril morphology provided additional data for classifying three genera in separate tribes [26]. Furthermore, many authors studied *praecitrullus fistulosus* for its medicinal, anthelmintic, and anticancer activities [27,28,29]. Among the tendril anatomical characters presented in the study, only a few discussed characters have been studied earlier for selected species [20,22,23], while other species investigated in the current project have not been investigated. A detailed review of the literature revealed that there is no comprehensive study regarding the tendril anatomical features of these plants.

## 4. Materials and Methods

### 4.1. Study Site and Selected Species of Family Cucurbitaceae

This research was conducted in the desert areas of districts of Bhakkar and Layyah in Punjab province. During March to July 2022, 17 Cucurbitaceous species each with 5 specimens were collected during field trips. Cucurbitaceous species sampling sites were georeferenced using a GPS device (German eTrex Venture) (Table 5).

The whole specimen was collected, including the tendrils, roots, stems, petioles, leaves, and flowers. Cucurbitaceous species were grown in cultivated field crops and wild places of the studied region. Plant specimens were pressed and dried in newspapers, identified and authenticated from the Herbarium of Pakistan (ISL). Cucurbitaceous species names were verified from the International Plant Name Index (www.ipni.org accessed on 9 October 2020) and Flora of Pakistan (www.eflora.org accessed on 9 October 2020). After preservation with ethanol and mercuric chloride solution the Cucurbitaceous herbarium specimens were deposited in the ISL herbarium.

### 4.2. Tendril Fixation

A solution of FAA (one part of 40% formaldehyde, 18 parts of 70% ethanol, and one part of glacial acetic acid) was used to fix mature tendrils for anatomical study for 12 h. They were moved for 2 h in 50% ethanol and then dipped into 70% ethanol for 2 h. Afterward, put in absolute ethanol at room temperature [22].

### 4.3. Histological Sectioning

Successive fixation of tendrils were sectioned using the standard technique outlined by [30] with several changes. Tendril sections were cleaned for about a minute, and any extra water was then removed by treating the sections with a series of alcohols ranging from 70% to 100%. The dehydrated pieces were then submerged in xylol for one hour to permeate the wax. At 60 °C, molten wax was used to preserve the tissues. Sections were transferred to cast using forceps and needles, and a cast was used for this. This wax-filled cast and its sections were chilled with cold water. The cast was afterward lifted out and processed for microtomes. A piece of around 15–20 µm was cut from half of the length from the base with the aid of a Shandon Microtome (Finesse 325). On a glass slide, these sections were moved, and egg albumen was scattered all over the slide. The slide was moved onto a hot plate and placed in an oven at 60 °C, where the wax expanded. The tissues were extracted from the wax using xylol for five minutes. Sections were washed thoroughly before being successively dehydrated with alcohol at concentrations of 100%, 90%, 80%, and 70%. For staining, fast green stain and Safranin O were applied. Slides were stained for 15 to 20 min before being cleaned with distilled water. Rehydration using a sequence of alcohol concentrations of 70%, 80%, 90%, and 100%, respectively was then performed. To make the slide better visible, xylol was employed. DPX mountant was placed on the slide for mounting, and the area received a cover slip. Each slide had a proper label and was dried [31].

### 4.4. Tendril Micromorphology

Slides were examined using a 40x-objective LM (OPTIKA Microscope, Italy). Digital cameras were used to capture photos of each sample under the 4, 10, and 40 objective lenses.

### 4.5. Light Microscopy

A reading sheet was used to record quantitative data. 10 to 15 readings of each species were taken under the Meiji (MT 4300H) LM at a magnification of 40×. For each species, minimum, maximum, mean and standard deviation values of various microanatomical parameters, including the length and width of the tendrils, the vascular bundles, collenchymatous cells, sclerenchyma cells, chlorenchyma cells and parenchyma cells, abaxial and adaxial epidermal cells, and the vessel elements, were calculated [32].

### 4.6. Statistical Analysis

The statistical SPSS 16.0 tool was used to analyze the corresponding average data for the measured values of tendril anatomical traits [33]. The effectiveness of the quantitative and qualitative features were evaluated using the UPGMA clustering analysis based on the Euclidean distance coefficient using PAST 4.03 software [34].

## 5. Conclusions

Through microscopic magnifications, the anatomical morphometry of tendril micromorphological features in Cucurbitaceous taxa exhibited variations. The resulting tendril micromorphology provides trustworthy traits that help identify different species. Accurate taxonomy will be achieved through the analysis of tendril characteristics. For taxonomic examination, it is crucial to consider the shape of the tendril outline, vascular bundle arrangements, sclerenchyma layers, and the morphology of the collenchyma and epidermal cells. The largest collenchyma cell was found in *Cucumis sativus* (27.85 µm), whereas the longest sclerenchyma cell was found in *Luffa acutangula* var. *amara* (38.05 µm). The findings show that the taxonomic identification of these species and their relationships will benefit from quantitative anatomical tendril features through clustering (UPGMA) analysis

## Figures and Tables

**Table 1:** Qualitative tendril anatomical characters of Cucurbitaceous species.

Sr No.	Cucurbitaceous Taxa	Epidermal Cell Shape	Collenchyma Cell Type	Chlorenchyma Cell Shape	Sclerenchyma Cell Shape	Parenchyma Cell Shape	Vascular Bundle Shape	Vascular Bundles	Tendril Outline in Transverse View
1.	*Citrullus colocynthis* (L) Schrad.	Rectangular and isodiametric	Angular	Rectangular to irregular	Tetragonal to polygonal	Polygonal	Oval	Subsidiary	Slightly oval
2.	*Citrullus lanatus* (Thunb.) Matsum.& Nakai	Rectangular	Angular	Irregular	Polygonal to irregular	Irregular	Elliptical	Subsidiary	Irregular, slightly v shaped
3.	*Cucumis melo* L.	Rectangular	Angular	Irregular	Irregular	Irregular	Irregular	Subsidiary	Irregular shaped
4.	*Cucumis melo subsp. agrestis* (Naudin) Pangalo	Oval to irregular	Angular	Irregular	Polygonal	Polygonal	Irregular	Subsidiary	4 angled
5.	*Cucumis melo var. flexuosus* (L.) Naudin	Rectangular, square, and isodiametric	Angular	Irregular	Tetragonal to polygonal	Polygonal	Irregular	Subsidiary	Irregular shaped
6.	*Cucumis melo* var.*cantalupensis* Naudin	Rectangular to square	Angular	Polygonal	Triangular to polygonal	Irregular	Irregular	Central	Irregular shaped
7.	*Cucumis sativus* L.	Rectangular	Angular	Rectangular	Tetragonal to polygonal	Irregular	Elliptical and irregular	Subsidiary	Star-shaped
8.	*Cucurbita maxima* Duchesne	Rectangular	Angular	Rectangular to polygonal	Irregular	Irregular	Dumbbell and irregular	Subsidiary	Irregular shaped
9.	*Cucurbita pepo* L.	Rectangular to square	Lamellar and angular	Polygonal	Irregular	Irregular	Irregular	Subsidiary	Six angled
10.	*Cucurbita pepo* var. *cylindrica*	Rectangular, square to polygonal	Lamellar and angular	Polygonal	Irregular	Irregular	Oval and irregular	Subsidiary	Slightly c shaped
11.	*Lagenaria siceraria* (Molina) Standl.	Rectangular	Lamellar	Rectangular to irregular	Irregular	Irregular	Rounded	Subsidiary	Irregular with hollow pith
12.	*Luffa acutangula* (L.) Roxb.	Irregular	Angular	Irregular	Irregular	Irregular	Rounded	Subsidiary	Irregular with hollow pith
13.	*Luffa acutangula* var. *amara* C.B.Clarke	Rectangular, square to polygonal	Angular	Polygonal	Polygonal and irregular	Irregular	Round and oval	Subsidiary	Irregular polygonal
14.	*Luffa cylindrica* (L) M.Roem	Rectangular to square	Angular	Hexagonal to polygonal	Tetragonal to polygonal	Irregular	Round and elliptical	Subsidiary	Six angled
15.	*Momordica charantia* L.	Rectangular to irregular	Angular	Pentagonal to polygonal	Irregular	Irregular	Rounded and elliptical	Subsidiary	4 angled
16	*Momordica balsamina* L.	Pentagonal to hexagonal	Angular	Rectangular to polygonal	Tetragonal to hexagonal	Pentagonal to irregular	Oval and irregular	Central	4 angled
17	*Praecitrullus fistulosus* (Stocks) Pangalo	Rectangular	Angular	Pentagonal to polygonal	Irregular	Irregular	Irregular	Subsidiary	Polygonal, slightly U shaped

**Table 2:** Quantitative analysis of tendril anatomy of Cucurbitaceous taxa.

Sr No.	Cucurbitaceous Taxa	No. Of Vascular Bundles	Epidermal Cell Layer	Collenchyma Cell Layer	Chlorenchyma Cell Layer	Sclerenchyma Cell Layer	Parenchyma Cell Layer	Vessel Elements
1.	*Citrullus colocynthis* (L) Schrad.	4	1	3–4	1–2	3–5	4	6
2.	*Citrullus lanatus* (Thunb.) Matsum.& Nakai	6	1	2–3	1	3–4	4	5
3.	*Cucumis melo* L.	5	1	3	1	3	4	8
4.	*Cucumis melo subsp. agrestis* (Naudin) Pangalo	5	1	3	1–2	2–3	4	8
5.	*Cucumis melo* var. *flexuosus* (L.) Naudin	7	1	3	2	2–3	3	5
6.	*Cucumis melo* var.*cantalupensis* Naudin	6	1	3	2	2–3	4	4
7.	*Cucumis sativus* L.	5	1	4	1	2–4	6	15
8.	*Cucurbita maxima* Duchesne	7	1	3	2	5	4	9
9.	*Cucurbita pepo* L.	6	1	6	2	3–4	4	7
10.	*Cucurbita pepo* var. *cylindrica*	7	1	4	2	3–4	3	9
11.	*Lagenaria siceraria* (Molina) Standl.	7	1	3–4	2–3	7–9	5	8
12.	*Luffa acutangula* (L.) Roxb.	8	1	5	1	6–8	3	8
13.	*Luffa acutangula* var.*amara* C.B.Clarke	12	1	6	2	6	2	9
14.	*Luffa cylindrica* (L) M.Roem	8	1	5	1	6	3	9
15.	*Momordica charantia* L.	7	1	5	1	4	3	9
16	*Momordica balsamina* L.	3	1	3–5	1	1	3–4	10
17	*Praecitrullus fistulosus* (Stocks) Pangalo	10	1	2–5	2	4	5	5

**Table 3:** Quantitative tendril anatomical data of Cucurbitaceous species.

Sr No.	Cucurbitaceous taxa	L x W	Tendril Diameter (µm)	Upper Epidermal Cell (µm)	Lower Epidermis Cell (µm)	Collenchyma Cell (µm)
1.	*Citrullus colocynthis* (L) Schrad.	L	314 − 326.25 = 321.65 ± 5.02	16.75 − 23.25 = 19.85 ± 2.57	16.75 − 22.50 = 19.65 ± 2.25	10.50 − 21.25 = 16.80 ± 4.74
		W	297 − 304.75 = 301.10 ± 2.77	12.25 − 17.75 = 14.95 ± 2.13	12.75 − 18 = 15.10 ± 2.19	4.25 − 16.75 = 11 ± 4.72
2.	*Citrullus lanatus* (Thunb.) Matsum.& Nakai	L	512.50 − 517.75 = 514.95 ± 2.18	15.75 − 25.25 = 20.90 ± 3.75	14.75 − 25.75 = 20.55 ± 4.93	16.25 − 23.25 = 19.80 ± 2.56
		W	250 − 258.75 = 253.50 ± 3.24	10 − 20 = 12.9 ± 4.14	10.25 − 20.50 = 13.55 ± 4.12	7.50 − 12.50 = 10.05 ± 1.97
3.	*Cucumis melo* L.	L	126.75 − 145.25 = 134.10 ± 7.44	10.50 − 21.75 = 16.20 ± 4.50833	10 − 20.75 = 15.55 ± 4.28442	12.00 − 21.25 = 16.20 ± 3.77
		W	122.75 − 138 = 130 ± 6.38	8.75 − 13.75 = 11.05 ± 2.07	8.75 − 13 = 10.6 ± 1.85	7.50 − 13.75 = 10.65 ± 2.75
4.	*Cucumis melo subsp. agrestis* (Naudin) Pangalo	L	200.50 − 212.50 = 205.35 ± 5	3.75 − 25.50 = 16.70 ± 9.13	4.75 − 25.50 = 12.85 ± 9.49	13.00 − 35.50 = 25.50 ± 9.78
		W	183.50 − 188.50 = 186.30 ± 2.13	3.75 − 18 = 11.55 ± 6.18	2.50 − 15.25 = 7.25 ± 4.97	8.25 − 25.50 = 15.45 ± 6.54
5.	*Cucumis melo var. flexuosus* (L.) Naudin	L	325.25 − 333 = 328.85 ± 3.18	13 − 50.25 = 23.15 ± 15.28	13.50 − 45.25 = 22.50 ± 12.90	11 − 28 = 18.50 ± 6.98
		W	250.25 − 267.75 = 257.30 ± 7.73	7.75 − 16.75 = 11.15 ± 3.81	8 − 14.25 = 10.75 ± 2.97	2.75 − 13.25 = 8.5 ± 3.83813
6.	*Cucumis melo* var*.cantalupensis* Naudin	L	250.50 − 256.50 = 253.75 ± 2.384	18.75 − 26.25 = 22.7500 ± ± 2.87	19.50 − 26.50 = 22.6500 ± 2.831	13.00 − 24.75 = 18.7000 ± 4.396
		W	225 − 231.25 = 227.60 ± 2.36	13.75 − 23.25 = 18.20 ± 3.40221	13.25 − 19.50 = 16.70 ± 2.558	12 − 14.25 = 13.1500 ± 0.91
7.	*Cucumis sativus* L.	L	524.75 − 531.25 = 527.40 ± 2.71	20.75 − 30.00 = 26.0500 ± 4.052	17.75 − 30.50 = 25.9500 ± 5.7047	23.25 − 33.50 = 27.8500 ± 3.7358
		W	225.00 − 238.25 = 229.40 ± 5.375	10.25 − 13.00 = 11.5500 ± 1.242	10.25 − 13.00 = 11.5500 ± 1.267	13.75 − 21.25 = 17.7000 ± 2.808
8.	*Cucurbita maxima* Duchesne	L	589.75 − 600.25 = 595.60 ± 3.9115	19.25 − 42.25 = 27.3000 ± 10.24	19.50 − 41.00 = 27.2000 ± 9.8256	12.25 − 20.00 = 16.8500 ± 3.5820
		W	486.25 − 494.75 = 489.60 ± 3.3241	9.25 − 20.00 = 14.2500 ± 4.10	9.75 − 19.75 = 14.4000 ± 3.91152	8.75 − 13.25 = 10.7000 ± 1.68077
9.	*Cucurbita pepo* L.	L	650.00 − 662.75 = 656.10 ± 4.735	8.75 − 19.75 = 13.9000 ± 4.211	8.00 − 20.00 = 13.5000 ± 4.53114	9.50 − 15.25 = 12.8500 ± 2.29538
		W	315.50 − 321.25 = 317.60 ± 2.1837	7.50 − 17.50 = 11.4500 ± 3.8786	7.25 − 18.00 = 11.3000 ± 4.298	7.25 − 14.75 = 10.4500 ± 3.0893
10.	*Cucurbita pepo* var. *cylindrica*	L	361.25 − 368.00 = 364.45 ± 2.6774	22.50 − 31.25 = 26.5000 ± 3.579	22.75 − 31.00 = 26.5500 ± 3.188	16.75 − 25.00 = 21.7500 ± 3.292
		W	162.75 − 170.25 = 1.67.00 ± 2.93	13.00 − 22.50 = 18.0500 ± 3.692	13.25 − 22.00 = 18.0000 ± 3.592	10.50 − 19.25 = 14.8500 ± 3.223
11.	*Lagenaria siceraria* (Molina) Standl.	L	471.25 − 487.75 = 477.85 ± 6.125	14.50 − 27.50 = 20.4500 ± 5.874	14.25 − 27.75 = 20.7000 ± 6.2759	22.50 − 30.50 = 27.7500 ± 3.172
		W	210.00 − 223.75 = 217.65 ± 5.641	9.75 − 13.00 = 11.1000 ± 1.526	9.50 − 13.00 = 11.1500 ± 1.61632	8.75 − 12.75 = 10.7000 ± 1.71756
12.	*Luffa acutangula* (L.) Roxb.	L	250 − 676.25 = 582.85 ± 186.37	8.50 − 20.50 = 15.05 ± 4.56002	7.25 − 20.50 = 14.7 ± 5.03550	13.00 − 28.50 = 21.20 ± 6.13
		W	251.75 − 270.25 = 258.85 ± 7.86	5.25 − 10.50 = 8.05 ± 1.89	5.00 − 10.25 = 7.75 ± 1.87	7.50 − 17.50 = 12.20 ± 3.7
13.	*Luffa acutangula var.amara* C.B.Clarke	L	451.25 − 480.50 = 472.45 ± 11.99	17.50 − 26 = 21.05 ± 4.19	17.75 − 27.50 = 21.3 ± 4.8	15.50 − 25 = 20.30 ± 3.83
		W	219.75 − 252.75 = 229.20 ± 13.64	12.50 − 17 = 14.4 ± 1.82	12.75 − 16.25 = 4.1 ± 1.47479	12.75 − 22.75 = 15.9500 ± 3.98
14.	*Luffa cylindrica* (L) M.Roem	L	401.25 − 413.75 = 406.2 ± 4.84897	11.25 − 15.75 = 13.0000 ± 1.677	11.50 − 15.75 = 13.0000 ± 1.6488	15.75 − 23.25 = 19.7000 ± 3.2948
		W	172.25 − 180.25 = 175.75 ± 2.915	8.00 − 15.25 = 11.6500 ± 2.637	8.25 − 15.25 = 11.7000 ± 2.51496	13.00 − 18.00 = 15.0500 ± 2.041
15.	*Momordica charantia* L.	L	350.00 − 357.75 = 353.25 ± 3.177	11.75 − 17.00 = 14.7500 ± 2.186	12.00 − 17.00 = 14.6000 ± 2.2262	16.25 − 20.00 = 18.2500 ± 1.3578
		W	160.75 − 167.25 = 164.55 ± 2.7064	8.75 − 11.00 = 9.9000 ± 0.96177	9.00 − 10.75 = 9.9500 ± 0.71589	9.25 − 11.75 = 10.5500 ± 1.10962
16	*Momordica balsamina* L.	L	122.25 − 124.0 = 123.05 ± 0.81777	8.75 − 16.25 = 13.3000 ± 2.9811	8.50 − 16.00 = 13.3000 ± 3.15436	14.00 − 25.50 = 19.4500 ± 5.5884
		W	112.25 − 114.00 = 112.95 ± 0.64711	7.75 − 12.25 = 10.3000 ± 1.6240	8.00 − 12.00 = 10.3500 ± 1.49583	12.75 − 14.75 = 13.8500 ± 0.89443
17	*Praecitrullus fistulosus* (Stocks) Pangalo	L	575.25 − 584.00 = 578.95 ± 3.49	22.50 − 32.75 = 28.0000 ± 4.172	23.50 − 33.00 = 27.6500 ± 4.207	17.25 − 26.25 = 22.2000 ± 3.858
		W	238.00 − 244.75 = 241.90 ± 2.7477	13.00 − 19.25 = 16.2500 ± 2.417	13.25 − 18.75 = 16.1000 ± 2.043	10.50 − 20.00 = 14.6000 ± 3.529

**Table 4:** Quantitative tendril anatomical features among Cucurbitaceous species.

Sr No.	Cucurbitaceous taxa	L x W	Chlorenchyma Cell (µm)	Sclerenchyma Cell (µm)	Parenchyma Cell (µm)	Vessel Elements(µm)	Vascular Bundle (µm)
1.	*Citrullus colocynthis* (L) Schrad.	L	31.75 − 50.25 = 40.50 ± 7.65	11.25 − 25 = 25 ± 5.76	50.50 − 84.75 = 66.25 ± 15.04	18.00 − 26.25 = 21.70 ± 3.52	135.50 − 188 = 161.55 ± 21.20
		W	12.00 − 19.25 = 16.05 ± 3.04	10.50 − 22.25 = 16.10 ± 4.73	47.00 − 77.25 = 63.35 ± 13.40	5.70 − 9.90 = 7.82 ± 1.69	74.25 − 90.75 = 81.40 ± 6.79
2.	*Citrullus lanatus* (Thunb.) Matsum.& Nakai	L	16.25 − 27.00 = 21 ± 4.25	10.75 − 27.75 = 22.40 ± 6.76	38.75 − 101 = 74.65 ± 28.14	25.50 − 37.50 = 29.05 ± 4.85	89.50 − 200.25 = 146.15 ± 48.33
		W	8.25 − 14.75 = 10.65 ± 2.56	8.50 − 21 = 16.10 ± 4.84	26.25 − 28.75 = 27.35 ± 0.96	15 − 25 = 18.30 ± 4.44	27 − 89.75 = 64.25 ± 29.42
3.	*Cucumis melo* L.	L	18.75 − 26.25 = 22.15 ± 2.98	16.75 − 28 = 22.10 ± 4.83	18 − 43.75 = 29 ± 11.15	11.25 − 16.25 = 13.75 ± 2.26	89 − 113.75 = 99.90 ± 10.29
		W	8.75 − 11.75 = 10.35 ± 1.23	9.75 − 22 = 14.90 ± 5.23	9.75 − 22 = 14.90 ± 5.23	8.50 − 11.25 = 9.55 ± 1.10	30.50 − 55.25 = 38.20 ± 9.97
4.	*Cucumis melo subsp. agrestis* (Naudin) Pangalo	L	15.50 − 25.25 = 20.60 ± 4.05	8.25 − 20.25 = 13.55 ± 4.54	20.50 − 77.75 = 53.55 ± 26.50	9.75 − 13.25 = 11.50 ± 1.47	47.25 − 97 = 74 ± 19.78
		W	7.75 − 10.25 = 8.9000 ± 1.24	2.75 − 18.50 = 9 ± 6.02	27.75 − 62.75 = 46.25 ± 13.21	5.25 − 10.25 = 7.90 ± 1.78	22.50 − 33.75 = 28.20 ± 4.25
5.	*Cucumis melo var. flexuosus* (L.) Naudin	L	13 − 41.25 = 25.65 ± 10.97	5.25 − 20.50 = 16.15 ± 6.39	23.25 − 112.50 = 61.50 ± 37.46	7.75 − 13.75 = 11.75 ± 2.60	75.25 − 163 = 117.10 ± 39.43
		W	10.50 − 20 = 14.35 ± 4.27	3.75 − 15.25 = 10.15 ± 4.71	15.50 − 55.25 = 33.50 ± 14.27	5.50 − 13.25 = 9.60 ± 3.02	26.75 − 51.50 = 42.55 ± 9.86
6.	*Cucumis melo* var.*cantalupensis* Naudin	L	42.00 − 46.25 = 43.80 ± 1.67	19.25 − 25.75 = 21.35 ± 2.61	49.25 − 86.25 = 68.10 ± 13.73	29.75 − 40.75 = 34.95 ± 4.48	77.25 − 106.25 = 89.10 ± 11.14
		W	24.50 − 29.50 = 27.45 ± 2.06	10.50 − 14.25 = 12.20 ± 1.47	36.25 − 52.75 = 44.15 ± 6.78	21.25 − 37.75 = 30.45 ± 6.66	26.25 − 37.25 = 30.95 ± 4.10
7.	*Cucumis sativus* L.	L	25.50 − 62.75 = 44.25 ± 13.87	10.25 − 30 = 19.05 ± 8.56	13.75 − 88 = 56.95 ± 32.34	38.25 − 47.50 = 42.55 ± 4.09	151.75 − 301.25 = 224.15 ± 56.12
		W	13.75 − 20.25 = 17 ± 2.54	7.75 − 20.25 = 14.25 ± 5.60	11.00 − 39.25 = 24.45 ± 10.77	20.50 − 38 = 29.25 ± 7.35	101.50 − 202.25 = 125.30 ± 43.31
8.	*Cucurbita maxima* Duchesne	L	20.50 − 51.25 = 33.60 ± 11.74	13.75 − 27.75 = 20.70 ± 5.67	24.75 − 64.50 = 45.15 ± 16.02	24.75 − 43 = 35.10 ± 6.83	30.50 − 38.75 = 34 ± 3.15
		W	11.75 − 18 = 13.55 ± 2.60	9.50 − 12.50 = 11.45 ± 1.19	14.50 − 28.25 = 20.80 ± 6.11	18.75 − 27.75 = 22.90 ± 3.92	24.75 − 30.75 = 27.35 ± 2.24
9.	*Cucurbita pepo* L.	L	21.75 − 29.25 = 26.60 ± 3.12	20.25 − 30 = 24.05 ± 3.68	30.50 − 66.25 = 53.35 ± 14.31	25.75 − 35.25 = 30.80 ± 3.69	37.25 − 44.50 = 41.55 ± 3.12
		W	7.25 − 19.75 = 10.95 ± 5.05	9.75 − 14.25 = 11.25 ± 1.76	17.75 − 50.75 = 30.95 ± 12.30	15.25 − 33.75 = 24.75 ± 8.65	24.50 − 29.50 = 27.55 ± 1.93
10.	*Cucurbita pepo var. cylindrica*	L	26.75 − 46.25 = 33.55 ± 7.57	19.50 − 25.25 = 23.05 ± 2.34	47.25 − 98.75 = 63.45 ± 20.67	16.25 − 22.25 = 19 ± 2.34	22.50 − 28.75 = 26.10 ± 2.87
		W	12.50 − 28 = 19.10 ± 7.40	12.25 − 18 = 16.35 ± 2.32	12.25 − 25 = 18.45 ± 5.13	11.25 − 15 = 13.30 ± 1.52	17.25 − 21.25 = 19.35 ± 1.51
11.	*Lagenaria siceraria* (Molina) Standl.	L	20 − 46.25 = 32.50 ± 10.08	22.50 − 31.25 = 27.60 ± 3.43	43.75 − 105.25 = 74.15 ± 22.10	20 − 37.50 = 28.30 ± 7.28	63.25 − 103.75 = 86.25 ± 17.20
		W	11.75 − 17.50 = 13.40 ± 2.38	11.75 − 20.25 = 15.05 ± 3.65	26.75 − 36.50 = 31.40 ± 4.32	18 − 30.75 = 24.80 ± 5.14	25.50 − 63.50 = 38.95 ± 14.65
12.	*Luffa acutangula* (L.)Roxb.	L	17.75 − 45.50 = 30.50 ± 10.42	15.75 − 35.75 = 26.70 ± 8.98	24.25 − 38.75 = 31 ± 5.76086	25.50 − 32.75 = 27.60 ± 3.04	76.25 − 125.25 = 109.85 ± 19.32
		W	11.25 − 15.25 = 13 ± 1.60	11.00 − 15.50 = 13.50 ± 1.81	10 − 15.25 = 13.50 ± 2.05	13.25 − 30 = 23.95 ± 6.43	46.50 − 101.75 = 87.60 ± 23.57
13.	*Luffa acutangula* var. *amara* C.B.Clarke	L	15.25 − 45 = 29.85 ± 10.89	30.75 − 46 = 38.05 ± 6.45	23.25 − 82.81 = 41.61 ± 24.37	12.75 − 25.25 = 17 ± 5.54	76.25 − 125.25 = 109.90 ± 19.33
		W	10.25 − 15.75 = 13.45 ± 2.25	13.00 − 22.75 = 17.95 ±3.97	12.75 − 20.75 = 16.40 ± 3.83±	10.25 − 22.75 = 15.80 ± 5.58	46.50 − 101.75 = 87.60 ± 23.57
14.	*Luffa cylindrica* (L) M.Roem	L	17.50 − 37.75 = 28.80 ± 7.97	21.25 − 31.25 = 26.50 ± 3.99	53 − 75.75 = 65.34 ± 9.45473	10.25 − 31.25 = 21.70 ± 9.30	101 − 139.25 = 119.90 ± 17.32
		W	11.00 − 20.50 = 15.60 ± 3.76	11.25 − 17.50 = 13.80 ± 2.68	19.50 − 45.25 = 38.50 ± 10.88	7.50 − 27.75 = 17.55 ± 8	76 − 101.75 = 84.75 ± 10.84
15.	*Momordica charantia* L.	L	31.25 − 53.50 = 45.20 ± 9.05	21.25 − 26.25 = 24.45 ± 2.01	30.25 − 41.75 = 37.20 ± 4.36	22.25 − 31 = 26.85 ± 3.83	95.50 − 112.75 = 102.90 ± 7.33
		W	11.25 − 28.50 = 19.35 ± 6.64	11.50 − 18 = 14.95 ± 2.63	11.25 − 19.50 = 15.05 ± 3.83	17.50 − 23.75 = 20.75 ± 2.89	71 − 77.25 = 73.70 ± 2.61
16	*Momordica balsamina* L.	L	16.25 − 28.75 = 22.45 ± 4.88	16.25 − 21.25 = 19.60 ± 1.94	38.75 − 64 = 52.05 ± 9.07	7.25 − 11 = 9.50 ± 1.57	51.25 − 68.75 = 59.15 ± 7.87
		W	13.75 − 20.50 = 16.70 ± 3.10	11.25 − 17.75 = 13.95 ± 2.58	31.25 − 48.75 = 39.95 ± 7.16	7.75 − 9.75 = 8.60 ± 0.741	41.25 − 50.50 = 45.90 ± 3.90
17	*Praecitrullus fistulosus* (Stocks) Pangalo	L	20.00 − 47.50 = 32.70 ± 11.01	23.25 − 31.25 = 27 ± 3.08	62.50 − 126.75 = 91.55 ± 26	13 − 22.75 = 18 ± 4.09	138.25 − 153.75 = 147 ± 6.25
		W	13.75 − 25.50 = 19.85 ± 4.97	12.25 − 17.75 = 14. ± 2.22	20 − 75 = 41.65 ± 27.69	10.25 − 18 = 14.35 ± 3	104.50 − 127.75 = 118 ± 11.78

**Table 5:** Cucurbitaceous plant sampling along with localities, GPS coordinates, and herbarium vouchering.

S/N	Species Name	Locality	GPS Coordinates	Collection Date	Voucher Number	Accession Number
1.	*Citrullus colocynthis* (L) Schrad.	Patti Bulanda	31°15′24.67” N 71°25′28.66” E	7 May 2022	QAU-NA-3	132045
2.	*Citrullus lanatus* (Thunb.) Matsum.& Nakai	Mian farm	31°17′46.31” N 71°24′41.17” E	19 March 2022	QAU-NA-16	132053
3.	*Cucumis melo* L.	Mian farm	31°17′46.31” N 71°24′41.17” E	19 March 2022	QAU-NA-15	132059
4.	*Cucumis melo subsp. agrestis* (Naudin) Pangalo	Rakhhonda lala	31°19′01.56” N 71°25′50.80” E	7 May 2022	QAU-NA-7	132049
5.	*Cucumis melo* var*. flexuosus* (L.) Naudin	Patti Bulanda	31°15′24.67” N 71°25′28.66” E	8 May 2022	QAU-NA-6	132047
6.	*Cucumis melo* var*.cantalupensis* Naudin	Rakhhonda lala	31°19′01.56” N 71°25′50.80” E	7 May 2022	QAU-NA-9	132046
7.	*Cucumis sativus* L.	Mian farm	31°17′46.31” N 71°24′41.17” E	19 March 2022	QAU-NA-14	132060
8.	*Cucurbita maxima* Duchesne	47 TDA	31°31′46.92” N 71°09′56.33” E	22 March 2022	QAU-NA-8	132052
9.	*Cucurbita pepo* L.	47 TDA	31°31′46.92” N 71°09′56.33” E	20 March 2022	QAU-NA-13	132056
10.	*Cucurbita pepo* var. *cylindrica*	Jahan khan	31°33′09.40” N 71°10′17.71” E	20 March 2022	QAU-NA-11	132051
11.	*Lagenaria siceraria* (Molina) Standl.	Jahan khan	31°33′09.40” N 71°10′17.71” E	20 March 2022	QAU-NA-17	132050
12.	*Luffa acutangula* (L.)Roxb	Basti Thind	31°15′31.94” N 71°27′20.64” E	22 March 2022	QAU-NA-2	132055
13.	*Luffa acutangula* var.*amara* C.B.Clarke	Darkhana wala	31°16′13.57” N 71°25′03.28” E	11 May 2022	QAU-NA-5	132044
14.	*Luffa cylindrica* (L) M.Roem	Basti Thind	31°15′31.94” N 71°27′20.64” E	10 May 2022	QAU-NA-10	132054
15.	*Momordica charantia* L.	Jahan khan	31°33′09.40” N 71°10′17.71” E	20 March 2022	QAU-NA-1	132057
16.	*Momordica balsamina* L.	222 TDA	31°09′43.53” N 71°12′38.90” E	14 July 2022	QAU-NA-18	132143
17.	*Praecitrullus fistulosus* (Stocks) Pangalo	Fateh pur	31°10′28.68” N 71°13′32.71” E	10 May 2022	QAU-NA-4	132048

## Data Availability

The data that support the findings of this study are available from the corresponding author upon reasonable request.
